# Annual effective dose from environmental gamma radiation in Bushehr city

**DOI:** 10.1186/2052-336X-12-4

**Published:** 2014-01-06

**Authors:** Ali Mahmoud Pashazadeh, Mahdi Aghajani, Iraj Nabipour, Majid Assadi

**Affiliations:** 1 The Persian Gulf Nuclear Medicine Research Centre, Bushehr University of Medical Sciences, Bushehr, Iran; 2 The Persian Gulf Tropical Medicine Research Centre, The Persian Gulf Biomedical Research Centre, Bushehr University of Medical Sciences, Bushehr, Iran

**Keywords:** Effective dose, Outdoor, Indoor, Background gamma radiation

## Abstract

**Background:**

Present study was an attempt to measure outdoor and indoor gamma dose rates in Bushehr city to determine corresponding annual effective dose and, to assess effect of active nuclear power plant located in Bushehr city on background radiation level of this city.

**Methods:**

All measurements were performed by G.M (Geiger Muller) detector (X5C plus) calibrated in Iran Atomic Energy Agency. In order to avoid effects of ground on outdoor and indoor measurements, G.M detector was placed one meter higher than ground level. Also, during the outdoor measurements, G.M detector was used at least six meters away from the walls of any building nearby to avoid unwanted effects of the materials used in the buildings on measurements.

**Results:**

Average gamma dose rates of outdoor and indoor measurements were determined as 51.8 ± 8.8 nSv/h and 60.2 ± 7.2 nSv/h, respectively. Annual effective dose due to background gamma radiation was calculated as 0.36 mSv which was lower than average global level.

**Conclusions:**

The average annual effective dose from background gamma radiation in Bushehr city was less than global level. Comparison of the results of present study, as follow up, with previous attempt performed in 2004 to determine effective dose of environmental gamma radiation in Bushehr province revealed that, during eight years, nuclear power plant located in this city has not significantly increased level of annual effective dose of Bushehr city.

## Background

One inescapable feature of life in the earth is exposure to ionizing radiation. Ionizing radiation of the environment is the most ubiquitous form of exposure therefore determination of health risk of background gamma radiation is of great importance in health physics [1]. The first step to achieve this goal is to measure background gamma radiation level of region. Results of this measurement can also be used as a reference value to evaluate effects of man-made radiations. The main sources of background radiation are cosmic radiation and terrestrial radiation [2]. Cosmic radiation includes energetic particles produced by spallation reactions in the outer space of the atmosphere which penetrate into the earth atmosphere and contribute as one of the main sources of background radiation. Interaction of these particles with atmosphere molecules may produce cosmogenic radionuclides. Long half lived cosmogenic radionuclides have formed terrestrial radionuclides which exist in air, soils, rocks, water and building materials. The terrestrial predominant radionuclides, with respect to absorbed dose in human, are ^232^Th, ^238^U and ^40^K. ^232^Th and ^238^U are head of decay series in which radionuclides of the chain contribute to human exposure and increase total radiation on earth [2]. Environmental natural radiation highly depends on geological and geographical features of a region and also to the materials used in buildings of that region. Therefore background radiation levels may differ in different geographic locations [2,3]. Due to main contribution to human exposure, background radiation has been studied widely worldwide. As a part of this attempt, some studies have been performed in different cities of Iran [3-7]. Results of these studies show that level of background gamma radiation vary widely for different geographical locations. In some cities average dose rate is higher than mean value reported by United Nations of Scientific Commission on Effects of Atomic Radiation, UNSCEAR 2000 [2].

In a study performed in 2004 by a multipurpose survey meter (RDS-110) to measure environmental gamma radiation in Bushehr province, absorbed dose rate due to background gamma radiation was in the range of 47.2 nSv/h to 61.8 nSv/h and corresponding mean effective dose rate was in the range of 0.29 mSv/y to 0.38 mSv/y [8]. Because of some concerns regarding contribution of nuclear power plant located in Bushehr city on giving rise to environmental radiation, recording background gamma radiation dose rates may be significant in terms of elucidating the current situation and providing scientific answer to possible concerns. Therefore in this study, as follow up to assess possible effect of nuclear power plant, background gamma radiation in Bushehr city was assessed using G.M detector (X5plus) according to standard approach.

## Methods

**Figure 1:**
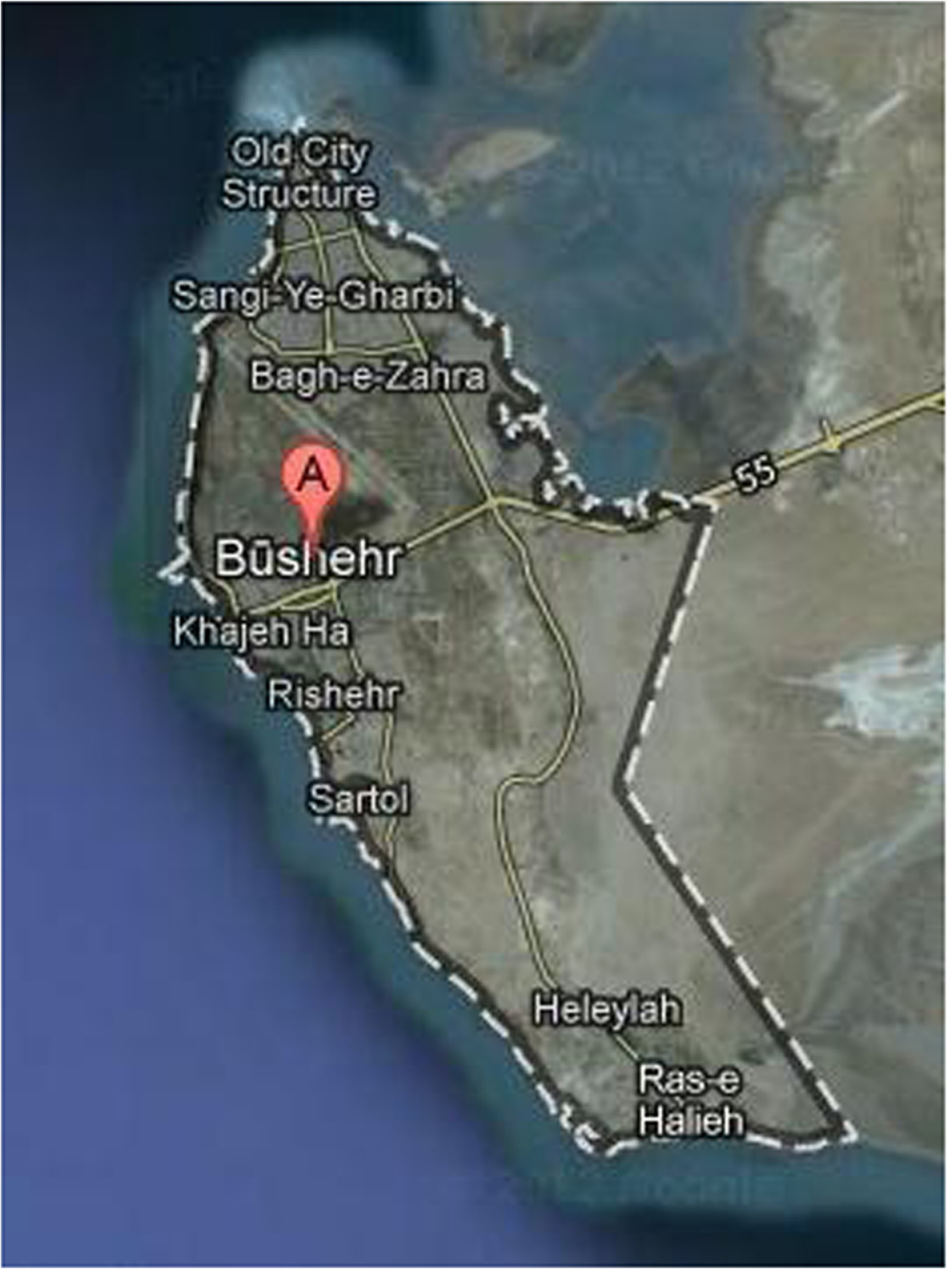
Map of Bushehr city (provided by Google Map).

Bushehr city is located in the south western part of Iran (28° 58′ 30″ N, 50° 50′ 17″ E) (Figure 1). In order to determine measurement sites in the city, using topographic map, Bushehr city was divided into five main areas as north, east, west, south and center of the city. For each of the areas, five stations and one building were randomly selected to measure outdoor and indoor background gamma radiation, respectively. Center of the city was assumed as a reference point for measurements then additional stations and sites were selected in north–south and east–west directions with an appropriate distance from each other.

Due to effect of type of buildings, buildings with similar and common used masonry materials were selected for measurements. Background gamma radiation measurements were performed by G.M detector (X5C plus), calibrated in Iran Atomic Energy Agency, during light day in October 2012. In order to avoid effects of ground and buildings on outdoor measurements, detector was used one meter higher than ground level and at least six meters away from the walls of any building nearby [4,9]. Also, detector was placed one meter higher than ground level inside the buildings to satisfy criteria for indoor measurements. For each measurement, the total exposure time of 1 hour was considered [5]. Mean of all measurements in each station and each building were computed and considered as outdoor and indoor absorbed dose of that station. Using calculated absorbed doses, annual effective dose from background gamma radiation was estimated as follow [2]:

$$E=\left(D_{\mathrm{out}}\times {\mathrm{OF}}_{\mathrm{out}}+D_{\mathrm{in}}\times {\mathrm{OF}}_{\mathrm{in}}\right)\times T\times \mathrm{CC}$$

Where E (nSv) is annual effective dose, D_out_ and D_in_ (nSv/h) are mean outdoor and indoor absorbed dose rates, T (hr) is time to convert from year to hour (8760 hours), OF_out_ and OF_in_ are outdoor and indoor occupancy factors (20% and 80% for outdoor and indoor, respectively) and CC is conversion coefficient (0.7 for adults) reported by UNSCEAR to convert absorbed dose in air to the effective dose in human [2].

## Results

Outdoor and indoor gamma dose rates and corresponding effective dose rates in five selected areas of Bushehr city are presented in Table 1. For each selected area, mean of the measurements and corresponding SD (standard deviation) were calculated. Maximum and minimum gamma dose rates were 57 ± 10 nSv/h and 46 ± 6 nSv/h for outdoor measurements and 69 ± 8 nSv/h and 51 ± 9 nSv/h for indoor measurements. Average outdoor and indoor gamma dose rates were determined as 51.8 ± 8.8 nSv/h and 60.2 ± 7.2 nSv/h, respectively. Ratio of indoor to outdoor gamma dose rate was determined as 1.16. Using average outdoor and indoor dose rates of environmental gamma radiation, annual effective dose of adults from background gamma radiation in Bushehr city was calculated as follow:

$$\begin{array}{l} E=\left(60.2\times 0.8+51.8\times 0.2\right)\times 8760\times 0.7\\ \;=0.36\;\mathrm{mSv} \end{array}$$

**Table 1:** Average outdoor and indoor gamma dose rates and resulted effective dose in selected areas of Bushehr city

**Area**	**Mean indoor dose rate (nSv/h) ± SD**	**range**	**Mean outdoor dose rate (nSv/h) ± SD**	**Range**	**Effective dose rate (mSv/y) ± SD**
North	54 ± 6	39-67	46 ± 8	37-71	0.32 ± 0.04
East	58 ± 7	43-78	57 ± 10	32-94	0.35 ± 0.05
West	69 ± 6	50-87	55 ± 11	30-78	0.41 ± 0.04
South	51 ± 9	34-75	51 ± 7	32-68	0.31 ± 0.05
Center	68 ± 8	48-94	49 ± 7	29-68	0.39 ± 0.05

## Discussion

Background gamma dose rates (outdoor and indoor) and corresponding annual effective dose were determined for Bushehr city. The results of this study, obtained by a G.M detector, indicated that effective dose from background gamma radiation in Bushehr city was less than global level. On the other hand, an adult individual living in Bushehr city receives an effective dose of 0.36 mSv from environmental gamma radiation each year which is lower than 0.48 mSv reported by UNSCEAR [2]. Due to geographical features, background gamma radiation in Bushehr city is among the low background radiation levels.

Based on the results of this study variation in our measurements, in comparison to similar studies, was relatively low. While wide variation was reported in gamma dose rates of different locations in other studies [4,5]. The main reason for this difference is variation in the geographical features of the areas. Because of the geography of the Bushehr city, there was not significant variation in the altitude and latitude of the specified sampling sites therefore wide variation was not observed in measurements (46 to 57 nSv/h for outdoor measurements and 51 to 69 nSv/h for indoor measurements).

Altitude and latitude are two determining factors on background radiation level [2-5,7,9]. According to the results of the studies, there is a linear function between altitude and annual effective dose from background gamma radiation [3]. Effective dose due to background gamma radiation in high altitude regions such as Azarbayjan (0.88 mSv), khorasan South (0.68 mSv), Khorasan Razavi (0.70 mSv), Khorasan North (0.68 mSv), Kordestan (0.69 mSv) and Lorestan (0.72 mSv) (higher than 1000 meters) is more than in low altitude regions such as Bushehr (0.36 mSv) and Hormozagnam (0.30 mSv) (approximately at sea level) [3,4,10,11]. One of the reasons is low amount of neutrons in regions with low altitude. At low altitude regions, neutron component of the cosmic ray can’t penetrate deeply into the atmosphere to reach to the ground. Maximum dose of neutrons is at the altitude of 10–20 km above the ground and decreases rapidly to small amount at sea levels [2]. Also, due to attenuation effects of atmosphere layers, directly-ionizing component of the cosmic ray is more attenuated at lower altitudes. In conclusion, background radiation decreases as altitude of the region decreases.

It is also indicated that there is a direct relationship between background dose rate and latitude of the region [2,4,12]. Bushehr city, located in south western part of Iran, has lower background gamma radiation in comparison to higher latitude regions of Iran [3,13]. One main reason for this phenomenon is magnetic field of the earth which increases by latitude and reach to the optimum value at poles. Magnetic field of the earth can affect slow moving charged particles and diverts them towards the poles. Comparison of the results of similar studies in different regions of Iran shows that effective dose of background gamma radiation increases with latitude up to 35°north [3].

One important finding of present study was revealed when our result was compared with result of previous study performed in 2004 to measure annual effective dose from background gamma radiation in Bushehr province. We found out that in former study annual effective dose rates from background gamma radiation were in the range of 0.29 mSv/y to 0.38 mSv/y and mean effective dose rate was determined as 0.35 mSv/y. comparison of results of these two similar studies in two different times revealed that background gamma radiation level of Bushehr city has almost not changed during eight years. This is of particular importance because of active nuclear power plant located in Bushehr city. Based on this comparison, activation of nuclear power plant during eight years from 2004 to 2012 hasn’t had significant effect on the level of background gamma radiation level of Bushehr city.

## Conclusions

The average annual effective dose from background gamma radiation in Bushehr city was 0.36 mSv/y, which is less than global level. Comparison of the results of present study, as a follow up, with results of previous similar study performed in 2004, revealed that nuclear power plant has not significantly affected background gamma radiation level in Bushehr city during eight years.

## Competing interests

The authors declare that they have no competing interests.

## Authors’ contributions

A. Mahmoud Pashazadeh developed initial idea and supervised the whole study. M. Aghajani involved in data collection. M. Assadi and I. Nabipour helped to revise manuscript. All authors read and approved the final manuscript.

## References

[B1] UNSCEAR Sources and effects of ionizing radiation, annex B: exposure of the public and workers from various sources of radiation 2010 New York: United Nations Scientific Committee on the Effect of Atomic Radiations

[B2] UNSCEAR REPORT Vol. I sources and effects of ionizing radiation, annex a: dose assessment methodologies 2000 New York: United Nations Scientific Committee on the effects of atomic radiation

[B3] Bahreyni Toossi MT Bayani S Yarahmadi M Aghamir A Jomehzadeh A Parast MH Tamjidi A Gonad, bone marrow and effective dose to the population of more than 90 towns and cities of Iran, arising from environmental gamma radiation Iran J Radiat Res 2009 7 41 47

[B4] Gholami M Mirzaei S Jomehzadeh A Gamma background radiation measurement in Lorestan province, Iran Iran J Rad Res 2011 9 89 93

[B5] Hazrati S Baghi AN Sadeghi H Barak M Zivari S Rahimzadeh S Investigation of natural effective gamma dose rates case study: Ardebil Province in Iran Iran J Environ Sci Eng 2012 9 1 10.1186/1735-2746-9-1 PMC3555127 23369115

[B6] Saghatchi F Eslami A Salouti M Assessment of annual effective dose due to natural gamma radiation in Zanjan (Iran) Radiat Prot Dosimetry 2008 132 3 346 349 10.1093/rpd/ncn285 18987116

[B7] Shahbazi-Gahrouei D Annual background radiation in Chaharmahal and Bakhtiari province Iran J Radiat Res 2003 1 87 91

[B8] Tamjidi A Bahreyni Toossi MT An assessment of annual effective dose and sensitive organ dose from environmental gamma radiation in cities of Boushehr province 2004 Mashhad, Iran: The 6th Iranian Congress of Medical Physics

[B9] Shahbazi-Gahrouei D Natural background radiation dosimetry in the highest altitude region of Iran J Radiat Res 2003 44 285 287 10.1269/jrr.44.285 14646234

[B10] Bahreyni Toossi MT Sadeghzade AA Estimation of environmental gamma background radiation levels in Azarbayjan Iran J Basic Med Sci 2000 3 17 24

[B11] Bahreyni Toossi MT Jomehzadeh A Comparison of environmental gamma radiation of kerman province and indoor gamma dose rate in kerman city using thermoluminescent dosimeter (TLD) and RDS-110 Med J Hormozgan University 2005 9 3 173 180

[B12] Kam E Bozkurt A Environmental radioactivity measurements in Kastamonu region of northern Turkey Appl Rad Isot 2007 65 440 10.1016/j.apradiso.2006.11.005 17207627

[B13] Bouzarjomehri F Ehrampoush M Gamma background radiation in Yazd province; a preliminary report Iran J Rad Res 2005 3 17 20

